# Predictors of drop-out in a multi-centre longitudinal study of participation and quality of life of children with cerebral palsy

**DOI:** 10.1186/1756-0500-5-300

**Published:** 2012-06-15

**Authors:** Heather O Dickinson, Marion Rapp, Catherine Arnaud, Malin Carlsson, Allan F Colver, Jérôme Fauconnier, Alan Lyons, Marco Marcelli, Susan I Michelsen, Jackie Parkes, Kathryn Parkinson

**Affiliations:** 1 Institute of Health and Society, Newcastle University, Royal Victoria Infirmary, Newcastle upon Tyne, NE1 4LP, UK; 2 Klinik für Kinder und Jugendmedizin, Universitätsklinikum Schleswig-Holstein, Ratzeburger Allee 160, Lübeck, 23538, Germany; 3 Inserm, UMR 1027, Toulouse, F-31073, France; 4 The Queen Silvia Children’s Hospital, Göteborg University, Göteborg, S-41685, Sweden; 5 SIIM-Pole Exploitation, Université Joseph Fournier, CHU de Grenoble BP 217, Grenoble cedex, 9 38043, France; 6 Enable Ireland, Lavanagh Centre, Ballintemple, Cork, Ireland; 7 Azienda Sanitaria Locale Viterbo, Viale Trento 18 H, Viterbo, 01100, Italy; 8 National Institute of Public Health, Oster Farimagsgade 5, Copenhagen, 1353, Denmark; 9 School of Nursing & Midwifery, Queen’s University Belfast, 21 Stranmillis Road, Belfast, BT9 5AF, UK; 10 Institute of Health and Society, Newcastle University, William Leech Building, Newcastle upon Tyne, NE2 4HH, UK

**Keywords:** Attrition, Longitudinal study, Cerebral palsy

## Abstract

**Background:**

SPARCLE is a study across nine European regions which examines the predictors of participation and quality of life of children with cerebral palsy. Children and their families were initially interviewed in 2004/2005 when the children were aged 8–12 years (SPARCLE1); they were approached again in 2009/2010 at age 13–17 years (SPARCLE2). The objective of this report is to assess potential for bias due to family non-response in SPARCLE2. Logistic regression was used to assess whether socio-demographic factors, parental stress and child impairment were related to non-response, both overall and by category (failure to trace families, death of child, traced families declining to participate).

**Results:**

Of the 818 families who participated in SPARCLE1, 224/818 (27%) did not participate in SPARCLE2. 51/818 (6%) were not traced. Among the 767 traced families, 32/767 (4%) children with cerebral palsy had died, seven children had been incorrectly diagnosed as having cerebral palsy, thirteen families had moved out of the region and one family had language problems. Of the remaining 714 families, 120/714 (17%) declined to participate. Drop-out between SPARCLE1 and SPARCLE2 varied significantly between regions; families were more difficult to trace and more likely to decline to participate if the parents’ educational qualifications, as recorded in SPARCLE1, were lower; they were also more likely to decline to participate if SPARCLE1 recorded that they were more stressed or if they had not completed a SPARCLE1 stress questionnaire.

**Conclusions:**

To reduce the risk of bias, all SPARCLE2 analyses should allow for factors (region and walking ability) which determined the sampling strategy, either by adjusting for these factors or by using sampling weights. Further analyses should be performed, adjusting for additional factors that were associated with non-response: parents' educational qualifications, family structure and parental stress. To allow for differential non-response in studies which sample from population registers, such registers should routinely record socio-demographic information.

## Background

Participation in life situations and quality of life (QoL) are important aspects of people's wellbeing; for people with chronic conditions, they may be more relevant measures of their health than medical outcomes. Participation is an objective concept - what people actually do [1]; QoL is a subjective concept - how they perceive their lives [2]. Until recently, little was known about the participation and QoL of disabled children.

In 2004, the SPARCLE project, which was funded by the European Union, was set up to evaluate the influence of environment on the participation and QoL of children aged 8–12 years with cerebral palsy [3]. Children with cerebral palsy were studied because they have a range of cognitive and motor impairments and so are representative of the wider population of disabled children. SPARCLE sampled children from population-based registers in eight European regions; another region recruited children identified from multiple sources [3,4].

During adolescence, physical and psychological changes occur; although these may be more difficult for disabled than for able-bodied adolescents, little research has examined the lives of disabled adolescents. We therefore followed up, at age 13–17 years, the 818 children who had participated in SPARCLE, to identify what childhood and adolescent factors are associated with participation and QoL in adolescence [5]. We refer to the first and second waves of the study as SPARCLE1 and SPARCLE2 respectively.

Bias arises if the participants in a study are systematically different from the population of interest (the target population) [6]. This threatens the external validity of the study: it would be misleading to generalize findings from studies whose participants are not representative. Longitudinal studies are at particular risk of bias because this may arise not only when participants are initially selected but also when researchers try to follow them up [7].

We have already assessed the potential for bias due to family non-response in SPARCLE1 [4]. The aim of this report is to identify potential biases in SPARCLE2 due to non-response, both of SPARCLE1 participants and of additional families who had not participated in SPARCLE1 but who were targeted for SPARCLE2 in order to compensate for drop-out.

## Methods

### Sampling for SPARCLE1

The sample design has been described in detail elsewhere [3,4] and is summarised briefly below.

Children were eligible for SPARCLE1 if born between 31^st^ July 1991 and 1^st^ April 1997 and on population registers of children with cerebral palsy covering eight regions of six European countries that share a standardised definition and classification of cerebral palsy [8]: north England, Northern Ireland, west Sweden, east Denmark, southwest Ireland, central Italy, southeast France, southwest France. There were 1,884 such children. In regions with more than 200 registered children (north England, Northern Ireland, west Sweden, east Denmark), we sampled so that the number agreeing to participate would be between 100 and 120 with similar numbers of children at each level of severity; we did this by grouping children by walking ability and selecting random samples within strata in each region. In other regions, except southeast France, all eligible children were included; southeast France included only children born between September 1992 and December 1996. We sampled 1,174 eligible children of whom 743 (63%) took part [4]. A further region in northwest Germany recruited 75 children from multiple sources, using the same classification of cerebral palsy [8]; the age, gender and levels of impairment of these children were similar to those of eligible children recorded on the population-based registers [4]. Thus 818 children comprised the sample; the numbers in each region are shown in Table 1.

**Table 1:** Number of children sampled in SPARCLE2, by SPARCLE1 status, region and participation status in SPARCLE2

	**Population-based registers**																			
	North England*		Northern Ireland*		West Sweden*		East Denmark*		South west Ireland**		Central Italy**		**South east France**^**†**^		South west France**		**North west Germany**		**ALL REGIONS**	
																				
	**n**	*(%)*	**n**	*(%)*	**n**	*(%)*	**n**	*(%)*	**n**	*(%)*	**n**	*(%)*	**n**	*(%)*	**n**	*(%)*	**n**	*(%)*	**n**	*(%)*
***Longitudinal sample***																				
SPARCLE1 participants	**116**	*(100)*	**102**	*(100)*	**83**	*(100)*	**115**	*(100)*	**98**	*(100)*	**85**	*(100)*	**67**	*(100)*	**77**	*(100)*	**75**	*(100)*	**818**	*(100)*
Participated in SPARCLE2	80	*(69)*	85	*(83)*	68	*(82)*	77	*(67)*	*74*	*(76)*	*41*	*(48)*	*50*	*(75)*	*55*	*(71)*	64	*(85)*	594	*(73)*
Did not participate in SPARCLE2	36	*(31)*	17	*(17)*	15	*(18)*	38	*(33)*	*24*	*(24)*	*44*	*(52)*	*17*	*(25)*	*22*	*(29)*	11	*(15)*	224	*(27)*
***Supplementary sample***																				
(i) Children sampled in SPARCLE1, who																				
did not participate in SPARCLE1									**10**	*(100)*			**53**	*(100)*	**18**	*(100)*			**81**	*(100)*
Participated in SPARCLE2									*1*	*(10)*			*7*	*(13)*	*1*	*(6)*			9	*(11)*
Did not participate in SPARCLE2									*9*	*(90)*			*46*	*(87)*	*17*	*(94)*			72	*(89)*
(ii) Children not sampled in SPARCLE1	**60**	*(100)*	**22**	*(100)*			**33**	*(100)*	**5**	*(100)*	**1**	*(100)*	**38**	*(100)*	**22**	*(100)*	**10**	*(100)*	**191**	*(100)*
Late registrations	31		22				0		3		1		12		22				91	
Incomers	2		0				0		2		0		0		0				4	
On register for SPARCLE1 but not sampled	27		0				33		0		0		25	^†^	0				85	
Missed in SPARCLE1	0		0				0		0		0		1		0				1	
**Participated in SPARCLE2**	29	*(48)*	3	*(14)*			9	*(27)*	*2*	*(40)*	*1*	*(100)*	*8*	*(21)*	*2*	*(9)*	10	*(100)*	64	*(34)*
**Did not participate in SPARCLE2**	31	*(52)*	19	*(86)*			24	*(73)*	*3*	*(60)*	*0*	*(0)*	*30*	*(79)*	*20*	*(91)*	0	*(0)*	124	*(66)*
***All SPARCLE2 participants***	**109**		**88**		**68**		**86**		**77**		**42**		**65**		**58**		**74**		**667**	

* In these regions, a random sample of eligible children was targeted in SPARCLE1. ** in these regions, all eligible children were targeted. † Southeast France did not include in SPARCLE1 all registered children in the eligible range of dates of birth; this was partially rectified. in SPARCLE2.

### Sampling for SPARCLE2

**Figure 1:**
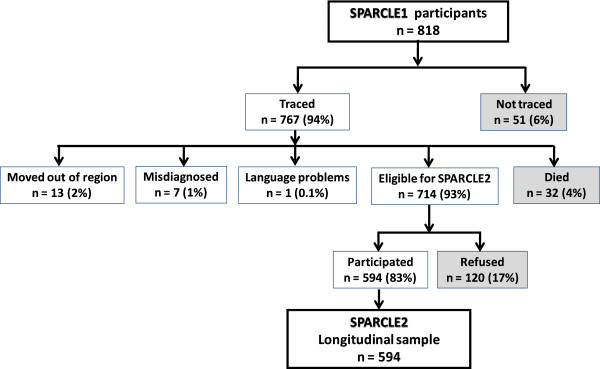
**Pattern of drop-out in longitudinal sample.** The denominator for each percentage is the number of families in the level immediately above. Shaded boxes indicate the categories of non-response that were analysed.

The families who responded to SPARCLE1 in 2004/2005 were followed up in 2009/2010 [5]. Those who responded comprised the *longitudinal sample* (see Figure 1 and Table 1).

**Figure 2:**
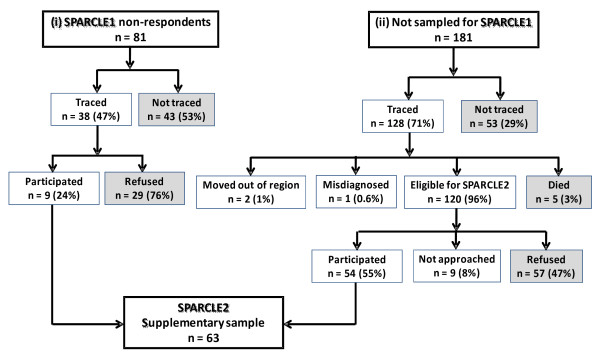
**Pattern of drop-out in supplementary sample.** Numbers exclude northwest Germany, which did not have a population-based register of children with CP. The denominator for each percentage is the number of families in the level immediately above. Shaded boxes indicate the categories of non-response that were analysed.

In order to maintain statistical power for cross-sectional analyses and possible further follow-up in adulthood, we additionally sampled adolescents who were eligible for SPARCLE1 but whose families had not participated in SPARCLE1 for various reasons (*supplementary sample***-** see Figure 2 and Table 1):

· Three regions (southwest Ireland, southeast France and southwest France) asked families who declined to participate in SPARCLE1 if they would be willing to participate in SPARCLE2 (see Table 1: supplementary sample (i)).

Eight regions (all regions except west Sweden) additionally approached families who had not been targeted for SPARCLE1 (see Table 1: supplementary sample (ii)):

· Six regions (north England, Northern Ireland, southwest Ireland, central Italy, southeast France, southwest France) approached families whose child was first recorded on the register after SPARCLE1 sampled: this group included both families resident in the region when SPARCLE1 sampled (late registrations) and families who moved into the region after SPARCLE1 sampled (incomers).

· Two regions (north England, east Denmark) had large registers and therefore for SPARCLE1 they had sampled from eligible children recorded on their registers; hence for SPARCLE2 they could additionally approach families of children who had been on their registers when SPARCLE1 sampled but had not been included in the SPARCLE1 sample.

· One region (southeast France) had restricted sampling of children for SPARCLE1 to those with dates of birth between September 1992 and December 1996; for SPARCLE2 they additionally approached families of children born between January and August 1992.

· Northwest Germany, which did not have a register, recruited ten additional families who had not participated in SPARCLE1.

The longitudinal sample and the supplementary sample together comprised the *cross-sectional sample.* None of the targeted families had more than one child with cerebral palsy.

### Interview of children and parents

Research associates visited children at home in 2004/2005 (SPARCLE1) to administer questionnaires to parents and children, if possible when the children were aged 8–12 years. Parents provided information about their type of employment and level of educational qualifications, whether the family lived in an urban or rural area, their child's age, gender, impairments (walking ability [9], fine motor skills [10], intellectual ability, vision, hearing, seizures, feeding, communication), school type, number and disability of siblings. Data on type of cerebral palsy type were available from the registers; in northwest Germany this was assessed by the research associates. The children's QoL, participation, environment, psychological health, pain and their parents' level of stress were recorded using a series of questionnaires [3,11-17].

In SPARCLE2, researchers visited families in 2009/2010 to recapture these characteristics, using the same (or slightly adapted) questionnaires or questionnaires more appropriate to self-report by adolescents [unpublished observations, C. Tuffrey].

To ensure quality control, the research associates from the different regions were trained together at a training workshop which included instruction in administering the questionnaires, engaging children, disability issues and the rationale for the study. Following this, each research associate carried out pilot visits in their own country. The research associates then met at a second workshop at which difficulties and dilemmas were discussed and clear decisions made to resolve them.

### Data quality

To ensure data quality, questionnaires were photocopied and sent to the co-ordinating centre where data were entered into an Access database. This was a continuous process from which centres received immediate feedback about omissions, ambivalent entries or inconsistency in their returns so that corrections could be submitted. Double data entry was then performed by an external company and discrepancies were checked and corrected. Data were then downloaded to Stata and further data checks were performed, including cross-validation of related data fields. As before, centres received feedback about inconsistencies so that data could be corrected.

All the analyses in this paper have an audit trail which may be inspected on request.

Researchers may apply to the SPARCLE group to undertake secondary analysis of SPARCLE1 data (see: http://www.research.ncl.ac.uk/sparcle). Equivalent access will be granted to SPARCLE2 data when the SPARCLE2 group have completed their primary analyses.

## Ethics approval and consent

The research complied with the requirement of the Helsinki Declaration. Ethics approval was obtained from NHS National Research Ethics Service (Newcastle and North Tyneside Research Ethics Committee, reference 09/H0906/4). All parents gave written consent; all children with sufficient cognitive capacity gave written consent or communicated consent if unable to write [3,5].

### Statistical methods

The objectives were:

a) to assess whether drop-out of SPARCLE1 participants varied by region and by child and family socio-demographic characteristics, as recorded in SPARCLE1;

b) to assess whether non-response of those families selected for SPARCLE2 who had not participated in SPARCLE1 varied with the level of impairment of the child, as initially recorded on the register;

c) to generate sampling weights that allowed for the sampling strategy and non-response;

d) to assess whether adolescents participating in SPARCLE2 in northwest Germany, where a population-based register of children with cerebral palsy was not available, were similar to adolescents with cerebral palsy in other regions in terms of age, gender and level of impairment.

These objectives were addressed as follows*:*

### A) Analysis of drop-out between SPARCLE1 and SPARCLE2

We considered all 818 families who participated in SPARCLE1. Non-response was categorised as due to (i) failure to trace the family (non-traceability), (ii) traced families declining to participate (refusal), and (iii) death of the child. As possible predictors of non-response (overall and by category), we considered factors recorded in SPARCLE1: characteristics of both the child (age, gender, types and levels of impairments, type of school attended) and of their family. Family characteristics included: family structure (parents: married living with partner, unmarried living with partner, single living with parents, single living alone); siblings (none, one or more but none disabled, one or more with some disabled); parental employment; parental educational qualifications; and parental level of stress as measured by the total stress score from the Parental Stress Index - Short Form questionnaire [16]. Statistically significant predictors were identified using logistic regression, stratified by region. Forwards stepwise regression followed by backwards steps was used to select covariates to enter into the model. The p-value for entry was 0.05 but, to lessen the probability of chance findings due to multiple hypothesis testing, the p-value for removal of covariates was set at 0·01. Initial models, considering each covariate in turn, excluded families with missing values on any of the covariates considered; the final multivariable models excluded only those families with missing values on the included covariates. All covariates were treated as categorical variables; parents' total stress was divided into quartiles, with missing values treated as a separate category. Adjacent categories were combined if their levels of non-response were not significantly different. Odds ratios (ORs) and their 95% confidence intervals (CIs) are reported. For categorical variables with more than two categories, these 95% confidence intervals were calculated from quasi-variances [18]; these confidence intervals allow valid comparison of odds ratios in any two categories whereas conventional confidence intervals only allow a valid comparison of each category with the reference group.

### B) Analysis of non-response in supplementary sample

We considered the supplementary sample: families who were eligible for SPARCLE1 but who had not participated in it and who were targeted for SPARCLE2. We excluded northwest Germany where participants were not selected from population-based registers. We used logistic regression, stratified by region, to assess whether non-traceability, refusal and overall non-response were related to age, or level of impairment (walking ability, presence of seizures, vision impairment) as recorded by the register when the child was first registered.

### C) Generation of sampling weights

Sampling weights were generated to allow for differential sampling and non-response in the SPARCLE2 longitudinal sample in different regions and in different levels of walking ability as recorded when the child was first registered. Children who had been misdiagnosed and children who had died between SPARCLE1 and SPARCLE2 were excluded and weights were calculated as the inverse of the probability of being a SPARCLE2 responder, which was estimated as the product:

prob(SPARCLE2 responder | SPARCLE1 responder, region, walking ability) X

prob(SPARCLE1 responder | in SPARCLE1 sample, region, walking ability) X

prob(in SPARCLE1 sample | region, walking ability)

Weights for the SPARCLE2 cross-sectional sample additionally allowed for the path whereby participants were recruited to the supplementary sample (SPARCLE1 non-participants or children not sampled in SPARCLE1). These weights, which weight the sample to correspond to the population of children with cerebral palsy recorded on registers in January 2004, will reduce bias in any analyses. The use of sampling weights inevitably increases the variance of estimates; this inefficiency was estimated using the Korn and Graubard statistic, which estimates the percentage increase in variance induced by using sampling weights and has a range from 0% (sampling weights do not increase the variance of estimates) to 100% (sampling weights yield extremely high variance of estimates) [19]. Weights for children in northwest Germany were set to one, as this region did not have a register.

### D) Comparison of German participants and others

We considered all adolescents who participated in SPARCLE2. We used logistic regression to compare adolescents in northwest Germany with those in other regions, on the basis of the age, gender and level of impairment as assessed at interview. We applied the weights generated above to the adolescents in other regions to ensure that they represented the target population of those regions.

Statistical analyses were performed using Stata 12 [20].

## Results

### A) Analysis of drop-out between SPARCLE1 and SPARCLE2

Figure 1 shows the pattern of drop-out of participants between SPARCLE1 and SPARCLE2. The multivariable logistic regression model showing the predictors of non-response, overall and by category, is presented in Table 2. Seven families were excluded from all analyses because their child had been incorrectly diagnosed as having cerebral palsy (see Figure 1). The number of additional children excluded because of missing data on covariates was seven in analysis of overall non-response, six in analyses of non-traceability and death and four in analysis of refusal.

**Table 2:** Multivariable logistic regression model relating drop-out between SPARCLE1 and SPARCLE2 to characteristics recorded in SPARCLE1

**Reason for drop-out:**	**(i) Not traced n/N = 51/805 (6%)**					(ii) Death n/N = 32/754* (4%)					(iii) Refusal n/N = 119/710**(17%)					**(iv) All non-response n/N = 214/804 (27%)**				
	n/N	(%)	OR	(95%CI)	p	n/N	(%)	OR	(95%CI)	p	n/N	(%)	OR	(95%CI)	p	n/N	(%)	OR	(95%CI)	p
Child impairment: Walking ability															0.005					
I. Walks without limitation											51/225	(22%)	1.0	(0.7 to 1.4)						
II. Walks with limitation											15/147	(10%)	0.3	(0.2 to 0.6)						
III. Walks with assistive devices											25/126	(20%)	0.8	(0.5 to 1.3)						
IV. Unable to walk, limited self-mobility											15/100	(15%)	0.5	(0.3 to 0.9)						
V. Unable to walk, severely limited self-mobility											13/112	(12%)	0.4	(0.2 to 0.8)						
Child impairment: Feeding										<0.001										
By mouth						14/700	(2%)	1.0	-											
By tube (partially or completely)						18/54	(33%)	9.1	(4.0 to 21)											
Information missing, excluded from analysis						0/1	(0%)	-	-											
Child impairment: IQ										<0.001										
≥50						4/533	(1%)	1.0	-											
<50						28/221	(13%)	9.2	(2.9 to 29)											
Information missing, excluded from analysis						0/5	(0%)	-	-											
Parental educational qualifications					0.002										0.001					<0.001
Above university entry	5/201	(2%)	0.3	(0.1 to 0.7)							25/190	(13%)	0.6	(0.4 to 0.9)		36/201	(18%)	0.4	(0.3 to 0.7)	
Intermediate	28/413	(7%)	1.0	(0.7 to 1.5)							57/359	(16%)	1.0	(0.8 to 1.3)		111/412	(27%)	1.0	(0.8 to 1.2)	
None or lowest formal qualifications	18/191	(9%)	1.4	(0.8 to 2.4)							37/161	(23%)	1.9	(1.2 to 3.0)		67/191	(35%)	1.7	(1.2 to 2.4)	
Information missing, excluded from analysis	0/6	(0%)	-	-							1/4	(25%)	-	-		3/6	(50%)	-	-	
Parental stress															<0.001					<0.001
Below 75th percentile											73/517	(14%)	1.0	(0.7 to 1.3)		138/581	(24%)	1.0	(0.8 to 1.3)	
Above 75th percentile											36/166	(22%)	2.2	(1.5 to 3.3)		60/190	(32%)	1.8	(1.3 to 2.5)	
Not recorded											10/27	(37%)	4.3	(1.8 to 10.3)		16/33	(48%)	3.6	(1.7 to 7.6)	
Family structure																				<0.001
Married, living with partner																135/567	(24%)	1.0	(0.8 to 1.2)	
Unmarried, living with partner																35/81	(43%)	2.7	(1.7 to 4.3)	
Single or separated and living with parents																10/18	(56%)	4.7	(1.7 to 13.0)	
Single, living alone																34/138	(25%)	1.1	(0.7 to 1.6)	
Information missing, excluded from analysis																0/1	(0%)	-	-	
Region					<0.001										<0.001					<0.001
North England	6/114	(5%)	1.0	(0.4 to 2.3)							22/102	(22%)	1.0	(0.6 to 1.7)		34/114	(30%)	1.0	(0.6 to 1.5)	
Northern Ireland	3/101	(3%)	0.5	(0.2 to 1.6)							8/93	(9%)	0.3	(0.2 to 0.7)		16/101	(16%)	0.4	(0.2 to 0.7)	
West Sweden	1/81	(1%)	0.3	(0.0 to 1.9)							6/74	(8%)	0.4	(0.2 to 1.0)		13/81	(16%)	0.4	(0.2 to 0.8)	
East Denmark	4/113	(4%)	1.1	(0.4 to 3.1)							26/103	(25%)	2.1	(1.3 to 3.5)		36/113	(32%)	1.9	(1.2 to 3.0)	
Southwest Ireland	6/96	(6%)	1.3	(0.6 to 3.0)							12/85	(14%)	0.7	(0.3 to 1.3)		23/96	(24%)	0.8	(0.5 to 1.3)	
Central Italy	16/85	(19%)	5.3	(3.0 to 9.2)							22/63	(35%)	2.6	(1.5 to 4.5)		44/85	(52%)	3.3	(2.1 to 5.2)	
Southeast France	8/67	(12%)	3.0	(1.4 to 6.3)							7/57	(12%)	0.5	(0.2 to 1.2)		17/67	(25%)	0.9	(0.5 to 1.7)	
Southwest France	6/74	(8%)	1.9	(0.8 to 4.5)							10/63	(16%)	0.7	(0.4 to 1.5)		21/73	(29%)	0.1	(0.7 to 1.9)	
Northwest Germany	1/74	(1%)	0.2	(0.0 to 1.8)							6/70	(9%)	0.3	(0.1 to 0.8)		10/74	(14%)	0.3	(0.2 to 0.7)	

n = number of non-respondents; N = total number analysed; % = percentage non-responders; OR = odds ratio; CI = confidence interval; p = likelihood ratio test statistic p-value for removal of characteristic from model; OR above 1.0 indicate a greater risk of non-response in that category than in the reference category.

For variables with more than two categories, 95%CI were based on quasi-variances [18].

* The denominator for analysis of deaths was all traced families.

** The denominator for analysis of refusal was all traced families with live children, excluding those who had moved out of the region (13 families) or who had language problems (1 family).

#### Non-traceability

Of the 818 families who participated in SPARCLE1, 51/818 (6%) families could not be traced (see Figure 1). The rate of failure to trace families varied significantly between regions, from 1% in west Sweden and northwest Germany to 19% in central Italy. Parents with lower educational qualifications were significantly less likely to be traced: compared with those with intermediate qualifications, the odds ratios for non-traceability among those with the highest and those with the lowest qualifications were 0.3 (95%CI: 0.1 to 0.7) and 1.4 (95%CI: 0.8 to 2.4) respectively (see Table 2).

#### Death of the child

Among the 767 traced families, 32/767 (4%) did not participate because their child had died (see Figure 1). The death rate did not vary significantly between regions. Children who were more severely impaired in terms of walking ability, fine motor skills, seizures, feeding and communication ability and IQ were significantly more likely to die. These impairments were highly correlated so, after allowing for feeding ability and IQ, which were the strongest predictors of death, the other impairments were not significant. Children who were fed by tube and those with an IQ below 50 were more likely to die, (OR = 9.1 (95%CI: 4.0 to 21) and OR = 9.2 (95%CI: 2.9 to 29) respectively, see Table 2).

#### Refusal to participate

The children who had died and families who had moved out of the region or had language problems were excluded from analysis of refusal, as their participation was not sought (see Figure 1). Among the remaining 714 families who were eligible for SPARCLE2, 120/714 (17%) did not wish to participate. Refusal rates varied significantly between regions, from 8% in west Sweden to 35% in central Italy. The severity of impairment of the child's walking ability, the parents' educational qualifications and parental stress were also significant predictors of refusal (see Table 2). Parents of children who walked without limitation or who walked with assistive devices were more likely to decline to participate. Parents with lower educational qualifications were also less likely to participate. Additionally, parental stress, as measured in SPARCLE1, was significantly associated with refusal: parents in the highest quartile of stress were more likely to decline to participate than those in the three lower quartiles (OR = 2.2 (95%CI: 1.5 to 3.3)); if the parents had not completed the stress questionnaire in SPARCLE1, they were even more likely to decline (OR = 4.3 (95%CI: 1.8 to 10.3)).

#### Overall non-response

Of the 818 families who participated in SPARCLE1, 224 (27%) did not take part in SPARCLE2. The non-response rate varying(see Table 2). Overall non-response varied significantly (p < 0.001) between regions, from 52% in central Italy to 14% in northwest Germany. It also varied with parental educational qualifications and parental stress, reflecting the findings for non-traceability and refusal (see Table 2). It also varied significantly (p < 0.001) with family structure, families being less likely to participate if parents were unmarried but living together and if parents were single and living with their own parents. This significant variation reflected similar but non-significant variations between these categories in non-traceability, refusal and death.

### B) Analysis of non-response in supplementary sample

This analysis excluded northwest Germany, which did not maintain a population register of children with cerebral palsy but recruited ten additional children from multiple sources. Figure 2 shows the pattern of non-response in the supplementary sample for all other regions.

#### SPARCLE1 non-responders

Three regions (southeast France, southwest France and southwest Ireland) attempted to interview for SPARCLE2 families whose children were sampled for SPARCLE1 but who did not participate in that wave of the study (see Table 1: supplementary sample (i)) Of the 81 families targeted, 43/81 (53%) were untraceable (of whom 30 had been untraceable in SPARCLE1) and among those traced 29/38 (76%) did not wish to participate (see Figure 2). Hence nine of the original 81 families targeted (11%) agreed to participate.

#### Families not sampled for SPARCLE1

Among the eight regions with population-based registers, seven regions targeted for SPARCLE2 a total of 181 families whose children were not sampled for SPARCLE1 (see Table 1: supplementary sample (ii)). These families had not been targeted in SPARCLE1 because: although they had been living in the region when SPARCLE1 sampled, their child was not recorded on the register until later (92 families), they had moved into the regions after SPARCLE1 sampled (4 families), their region had a large register which had allowed sampling of children for SPARCLE1 but their child had not been selected into that sample (60 families), or their region had failed to include in SPARCLE1 all registered children in the eligible range of dates of birth and this was partially rectified in SPARCLE2 (25 families). Among the 181 families targeted, 53/181 (29%) were untraceable (see Figure 2). Among the 128 traced families, 8/128 (6%) were not eligible because: they had moved out of the region (2 families), their child had been incorrectly diagnosed as having cerebral palsy (1 child), or had died (5 children). Of the remaining 120 eligible families, 9 (8%) were not approached and 57/120 (47%) declined to participate. Hence 54 of the original 181 families targeted (30%) agreed to participate.

#### All targeted families

In the combined supplementary sample of 262 eligible families targeted in these eight regions (81 SPARCLE1 non-respondents and 181 new families), 63 agreed to participate. Logistic regression showed no evidence that non-traceability, refusal of traced families, or overall non-response varied significantly (p < 0.01) with the adolescent’s age, or level of impairment (walking, vision, seizures) as recorded on the register when he or she was first registered.

### C) Generation of sampling weights

Sampling weights were calculated: the Korn and Graubard statistic, which estimates the percentage increase in variance introduced by using weights, was 33% for the longitudinal sample of 594 families and 75% for the cross-sectional sample of 667 families.

### D) Comparison of German participants and others

Northwest Germany had recruited 75 families for SPARCLE1, of whom 64 (85%) agreed to participate in SPARCLE2; ten additional families who had not participated in SPARCLE1 were recruited for SPARCLE2 (see Table 1). These 74 adolescents in northwest Germany were similar to the target population for SPARCLE2 in other regions in terms of level of impairment and gender, but they were significantly younger (p = 0.002 for age as a continuous variable) than those in other regions and, in particular, included a higher proportion of 12-year-olds (20% *vs.* 5%). This was largely because northwest Germany joined SPARCLE1 later than other regions and so the children recruited there tended to have later dates of birth to ensure they were within the prescribed age range for SPARCLE1. Similar results were obtained when analysis was restricted to the longitudinal sample.

## Discussion

### Summary of main findings

Of the 818 families who participated in SPARCLE1, 594 (73%) participated in SPARCLE2. The attrition of 27% between SPARCLE1 and SPARCLE2 was higher than the anticipated rate of 20% [5].

In order to maintain statistical power for cross-sectional analyses and possible further follow-up in adulthood, we had planned to approach 270 further families, anticipating a response rate of 63% as in SPARCLE1, which would have yielded 170 more participants [5]. However, of the 262 additional families who were targeted using population-based registers, only 63 (24%) agreed to participate, in marked contrast to the response rate of 63% in SPARCLE1 [4]. This disappointing response rate was partly due to targeting 81 families who had been sampled for SPARCLE1 but who had not participated, either because they were untraceable or had declined to participate; only nine (11%) of these families participated in SPARCLE2.

Hence the final cross-sectional sample size was lower than in SPARCLE1, both overall (667 families in SPARCLE2 compared to 818 families in SPARCLE1) and in all regions. The poorer response rate in SPARCLE2 may be a consequence both of families with younger children having a greater propensity to participate in surveys than those with older children [6] and of a lower response rate in more recent years [21].

### Predictors of drop-out

The predictors of each category of non-response are relevant to the design of future surveys.

· Rates of tracing varied between regions and, overall, parents with higher educational qualifications were easier to trace. However, educational qualifications may be a surrogate for socio-economic status, which we did not record because of the difficulties in obtaining a measure that was valid in all countries in the study.

· Rates of refusal of traced families likewise varied between regions, parents with higher educational qualifications being more likely to agree to participate. Additionally, if parents had been more stressed when visited in SPARCLE1 or if they had not completed the stress questionnaire, they were more likely to decline to participate in SPARCLE2. Refusal rates varied with the level of the child's walking ability but showed no clear trend with severity of impairment, although parents of less impaired children were generally less willing to participate.

· Drop-out due to death was much more common among more severely impaired children, in particular those with feeding and cognitive problems.

Predictors of overall non-response are of interest in the analysis and interpretation of SPARCLE2. They reflected the predictors of the main categories of non-response: failure to trace and refusal to participate. Hence parental educational qualifications and region, which were associated with both these categories of non-response, were strong predictors of overall non-response. Parental stress was also a predictor of overall non-response, although it was a statistically significant predictor only of refusal and not of non-traceability. Parents who were living together but not married and parents who were single (or separated) and living with their own parents were also under-represented in SPARCLE2. This finding is difficult to interpret. It appeared to be due to a combination of factors which were not significant when considered separately: these groups tended to be more difficult to trace, more likely to decline to participate if traced, and their children were more likely to die. It is possible that a difficult family situation, for example a child having poor health, could lead not only to stress – which we found was associated with refusal to participate – but also to marriage break-down, precipitating parents to move in with other partners or with their own parents and hence becoming more difficult to trace.

Although families with more impaired children were more likely to drop out because the child died, severity of impairment was not a significant predictor of overall non-response, partly because death was not a major cause of non-response and partly because parents of living children were more willing to participate if their child was more impaired.

The significant predictors – parental education and stress, family structure and region – may also have been associated with non-response in SPARCLE1 and with non-response of new families approached in SPARCLE2. However, as minimal data about the children and their families were recorded on the registers, we were unable to demonstrate such associations.

### Representativeness of sample

Missing data may be classified as: Missing at Random (MAR), if the probability that an observation is missing depends only on observed values and not on missing values, *i.e.* the missing values behave like a random sample of all values within subclasses defined by the observed data; or Missing Not at Random (MNAR) [22]. A special case of MAR occurs if the missing values are a simple random sample of all data values; in this case, the data are referred to as Missing Completely at Random. For data that are MAR, statistical adjustment such as use of non-response weights can yield unbiased estimates of effects despite the missing data; for MNAR data, this is not possible. However, although it is often assumed that data are MAR, no way to directly test this assumption is available [23]. In the context of SPARCLE, the danger is that non-respondents may have a systematically different quality of life or a different level of participation from respondents, which would invalidate estimates that assume data are MAR. On the other hand, the strength of our analysis of drop-out between SPARCLE1 and SPARCLE2 is that SPARCLE1 provided a wealth of information that could be used to predict drop-out in SPARCLE2 and hence to facilitate estimates that would be valid under the MAR assumption.

The target population, who were selected at random from population-based registers in Denmark, France, Italy, Sweden and the UK, can be regarded as representative of children with cerebral palsy. We cannot be sure that the German participants, who were recruited in other ways because that region did not maintain a population-based register, constitute a representative sample. The distribution of impairment and gender did not differ significantly between the German participants and the target population in other regions but a high proportion of German participants were interviewed before they had reached the prescribed age.

### Use of sampling weights

Use of sampling weights is essential to estimate population prevalences from the sample. However, the primary objective of SPARCLE is to estimate associations between outcomes and explanatory variables. Sampling weights may or may not be required to produce unbiased estimates of these associations [7]. If an estimate is valid with or without weights, the weighted estimate will typically be less precise. In general, an unweighted estimate is valid if the simple linear regression model holds, *i.e.* if it is valid to assume homoscedasticity, no interactions between explanatory variables, no omitted predictors and the sampling rate does not depend on the outcome variable [7,24]. Therefore, it is essential to conduct the usual checks of any unweighted regression models.

In analyses of the longitudinal sample, sampling weights may be used in order to allow both for the sampling strategy, which varied between regions and levels of walking ability, and for the variation in non-response between regions; they are likely to increase the variance of estimates by about a third. Alternatively, analyses could be adjusted for region and walking ability. In calculating sampling weights, we were unable to allow for differential non-response according to parental educational qualifications, family structure and stress, as such weights would have resulted in an unacceptable increase in the variance of estimates. Additional analyses should therefore be performed, with and without adjusting for these variables, and the estimates compared. Such analyses should demonstrate the effect of any differential non-response, assuming that data are missing at random within cells defined by these variables [25].

The diversity of sources making up the cross-sectional group makes it doubtful that appropriate sampling weights can be used. Sampling weights would probably increase the variance of estimates by about three-quarters, reflecting the small numbers of families who entered SPARCLE2 by each route in the supplementary sample, so adjustment for factors that determined the sampling design and non-response – region, walking ability, parental educational qualifications, family structure and stress – may be preferable [19].

### Comparison with other studies

We compared our findings with those of other surveys that targeted specific families in order to conduct face-to-face interviews [6,26,27].

Foster reported that non-contact rates were higher if the head of the household was single; we found no such association, probably because we had a much smaller sample [6]. Goodman reported that non-contact rates were higher in areas of greater deprivation [26]; we did not have a measure of deprivation, but we did find higher non-contact rates among parents with lower educational qualifications, which may be correlated with living in a deprived area.

Both Foster and de Winter reported that refusal of traced families was higher if the head of the household had lower educational qualifications [6,27], consistent with our findings. Goodman reported that parent refusal rates were marginally higher in areas of greater deprivation and child refusal rates increased steadily with increasing deprivation [26]; we found refusal rates were higher if parents had lower educational qualification or were more stressed, factors which may be associated with greater deprivation. De Winter reported higher refusal rates if the child was a boy or had unsatisfactory school performance [27]; we found no effect for gender and we had no measure of the child's school performance, although this may be associated with parental stress. Groves and Couper reported higher refusal rates among single person households and in urban areas [21]; we found no such associations, which may either be due to our smaller sample size or to different determinants of refusal in Europe and the U.S.; they also reported lower refusal rates among households with children under five years old and among households with younger adults; such an effect could partly explain the higher refusal rate in SPARCLE2 than SPARCLE1.

## Conclusions

All SPARCLE2 analyses should either adjust for region and walking ability (which determined the sampling design), or perform an analysis using sampling weights, and compare the resulting estimates with those from analyses without such adjustment or weighting. Use of sampling weights will probably result in an acceptable increase in variance of estimates for longitudinal, but not cross-sectional, analyses. Ideally, additional analyses should also be performed to consider the effect of adjustment for other factors associated with non-response: parental educational qualifications, family structure and parental stress.

Our findings also have more general implications. Registers which are used as a sampling frame for surveys should routinely record socio-demographic information such as parental education or socio-economic status as these factors are likely to influence non-response; capturing this information would allow corrections to be made for missing responses. Surveys should over-sample from those with lower educational qualifications, in order to compensate for anticipated lower response rates in these groups. It is doubtful whether, in follow-up waves of a longitudinal study, it is worthwhile attempting to include those who were untraceable or who declined to participate in the first wave. Survey findings should be interpreted assuming that the most stressed participants are least likely to respond.

## Abbreviations

CI: Confidence interval; CP: Cerebral palsy; MAR: Missing at random; NMAR: Not missing at random; OR: Odds ratio; QoL: Quality of life; SPARCLE: the Study of Participation of Children with Cerebral Palsy Living in Europe.

## Competing interests

The authors declare that they have no competing interests.

## Authors' contributions

AC conceived the study, participated in its design and directed the project; KP, AL, CA, MC, JF, SM, JP, MM and MR managed the acquisition of data in each region; KP and HD managed the data, HD performed all statistical analyses and wrote the paper. All authors have given final approval of the version to be published.
